# Biased assembly of the nuclear pore complex is required for somatic and germline nuclear differentiation in *Tetrahymena*

**DOI:** 10.1242/jcs.167353

**Published:** 2015-05-01

**Authors:** Masaaki Iwamoto, Takako Koujin, Hiroko Osakada, Chie Mori, Tomoko Kojidani, Atsushi Matsuda, Haruhiko Asakawa, Yasushi Hiraoka, Tokuko Haraguchi

**Affiliations:** 1 Advanced ICT Research Institute Kobe, National Institute of Information and Communications Technology (NICT), Kobe 651-2492, Japan; 2 Japan Women's University, Tokyo 112-8681, Japan; 3 Graduate School of Frontier Biosciences, Osaka University, Suita 565-0871, Japan; 4 Graduate School of Science, Osaka University, Toyonaka 560-0043, Japan

**Keywords:** Ciliates, Live CLEM, Live cell imaging, Nuclear differentiation, Nuclear envelope, Nuclear pore complex

## Abstract

Ciliates have two functionally distinct nuclei, a somatic macronucleus (MAC) and a germline micronucleus (MIC) that develop from daughter nuclei of the last postzygotic division (PZD) during the sexual process of conjugation. Understanding this nuclear dimorphism is a central issue in ciliate biology. We show, by live-cell imaging of *Tetrahymena*, that biased assembly of the nuclear pore complex (NPC) occurs immediately after the last PZD, which generates anterior-posterior polarized nuclei: MAC-specific NPCs assemble in anterior presumptive MACs but not in posterior presumptive MICs. MAC-specific NPC assembly in the anterior nuclei occurs much earlier than transport of Twi1p, which is required for MAC genome rearrangement. Correlative light-electron microscopy shows that addition of new nuclear envelope (NE) precursors occurs through the formation of domains of redundant NE, where the outer double membrane contains the newly assembled NPCs. Nocodazole inhibition of the second PZD results in assembly of MAC-specific NPCs in the division-failed zygotic nuclei, leading to failure of MIC differentiation. Our findings demonstrate that NPC type switching has a crucial role in the establishment of nuclear differentiation in ciliates.

## INTRODUCTION

Ciliated protozoa are unique unicellular organisms in which two functionally and structurally distinct nuclei, a somatic macronucleus (MAC) and a germline micronucleus (MIC), exist in a single cytoplasm (Orias et al., 2011). The MIC is diploid and contains chromosomes that remain condensed and are not transcribed during vegetative growth, whereas the polyploid MAC exhibits active transcription during all cellular stages (Gorovsky and Woodard, 1969). Both MAC (Eisen et al., 2006) and MIC (http://www.broadinstitute.org/annotation/genome/Tetrahymena/MultiHome.html) genomes have been completely sequenced. These two nuclei have distinct cell cycles, with distinct cycles of DNA replication and nuclear division at different times within the same cell. Understanding the mechanisms that generate this nuclear dimorphism is one of the most fascinating unsolved biological problems (Goldfarb and Gorovsky, 2009; Karrer, 2012).

**Fig. 1.**
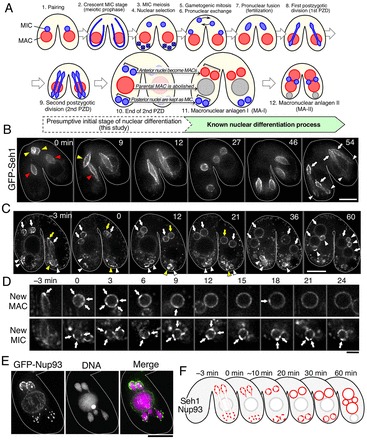
**Dynamic behaviors of GFP-tagged common Nups in conjugating *Tetrahymena* cells.** (A) Schematic representation of nuclear events during sexual reproduction. MICs and MACs are drawn in blue and red, respectively. Crescent MICs and post-zygotic nuclei undergo meiosis and mitosis, respectively. The nuclear envelope of these nuclei does not breakdown in meiotic and mitotic divisions. Parental (old) MACs (gray) are resorbed during nuclear differentiation (see also supplementary material Movie 1). (B) Typical time-lapse images of a conjugating pair of cells expressing GFP–Seh1 at stages from pronuclear exchange to the second PZD (cell numbers observed, *n* = 6 pairs). A set of 3D images (17 *z*-stacks×1.5 µm intervals) was taken every 3 min. The 3D images were projected after deconvolution and the images of the selected time points are shown (see also supplementary material Fig. S1). Yellow and red arrowheads indicate migratory and stationary pronuclei, respectively. White arrows and arrowheads indicate anterior and posterior regions, respectively, of dividing zygotic nuclei during the second PZD. The numbers represent the time in minutes after the start of observation. Scale bar: 10 µm. (C) Typical time-lapse images at stages from the second PZD to MA-II (in A) (*n* = 19 pairs). Images were taken as described in B except that 3D images (18 *z*-stacks×1.0 µm intervals) for each time point were taken. Arrows and arrowheads indicate presumptive new MACs and MICs, respectively. Yellow symbols indicate nuclei whose enlarged images are shown in D. Time 0 represents the time after the end of the second PZD. Scale bar: 10 µm. (D) Magnified views of the presumptive new MAC and MIC indicated by yellow symbols in C. Arrows indicate regions with strong fluorescence signals on the NE. Scale bar: 3 µm. (E) A conjugating pair of cells expressing GFP–Nup93 fixed at anaphase of the second PZD (*n* = 19 pairs). Upper- and lower sides are the anterior and the posterior regions of the cell, respectively. Scale bar: 10 µm. (F) Schematic diagram of the distribution of common Nup's (red). Time represents the time after the end of the second PZD.

A new MAC is generated from the MIC through sexual reproduction (conjugation). Once two cells with different mating types enter into conjugation, the MIC in each cell of the conjugating pair undergoes meiotic divisions to generate four haploid nuclei. One of these nuclei is then selected (the other three are degraded) and undergoes mitosis to generate one migratory and one stationary pronucleus. The migratory pronucleus is exchanged between conjugating partners and fuses with the stationary pronucleus of the other cell to form the zygotic nucleus; this process is equivalent to fertilization in higher eukaryotes. In *Tetrahymena*, two PZDs follow, the second of which is oriented along the long axis of the cell. The two nuclei in the anterior of the cell become new MACs and the two in the posterior differentiate into new MICs (Nanney 1953; Cole and Sugai, 2012; also see Fig. 1A).

One of the most striking events that occur during differentiation of the two nuclei is the rearrangement of the MAC genome. Within the MAC, MIC-specific internal eliminated sequences (IESs) are removed from the MAC genome during nuclear differentiation (Klobutcher et al., 1984; Yao et al., 1984). This event is mediated by small RNAs similar to those used for transcriptional gene silencing in RNA interference (Mochizuki et al., 2002; Mochizuki and Gorovsky, 2004). To accomplish this nuclear event, the components of the molecular machineries that are involved in DNA elimination, such as the Twi1p-siRNA complex, have to be transported into the correct nucleus with the correct timing (Chalker, 2008).

The nuclear pore complex (NPC) is of central importance in nuclear transport since proteins moving between the cytoplasm and the nucleoplasm must pass through the NPC. It is composed of multiple copies of about 30 nucleoporins (Rout et al., 2000; Cronshaw et al., 2002). In *Tetrahymena thermophila*, 13 nucleoporins have been identified (Iwamoto et al., 2009). Most components, including Seh1 and Nup93, are common to both MAC and MIC NPCs, but four Nup98 homologs are nucleus specific: MacNup98A and MacNup98B, possessing Gly-Leu-Phe-Gly (GLFG) repeats in their N-termini, are exclusively localized to the MAC, whereas MicNup98A and MicNup98B, possessing instead Asn-Ile-Phe-Asn (NIFN) repeats, are exclusively localized to the MIC (Iwamoto et al., 2009). Interestingly, the ability of MAC and MIC nuclear pore complexes to transport specific proteins differs (White et al., 1989), and these repeat sequences have a role in the localization of MAC- and MIC-specific nuclear proteins (Iwamoto et al., 2009). Thus, NPCs that contain MAC- and MIC-specific Nup98s homologs provide essential platforms for MAC- and MIC-specific nuclear transport.

The MAC- and MIC-specific composition and function of NPCs could explain, at least in part, ciliate nuclear dimorphism; however, there is no direct evidence that these nucleus-specific nucleoporins are involved in the process of nuclear differentiation. The zygotic nucleus is derived from parental MIC nuclei and is likely to contain MIC-type NPCs. Therefore, for the nuclei destined to become MACs to differentiate into mature MAC nuclei, MIC-type NPCs in these nuclei must be replaced with MAC-type NPCs. However, the dynamic behavior of NPC components during conjugation has never been studied in living cells. To observe dynamic processes of nuclear differentiation in *Tetrahymena*, we have developed technologies of fluorescence live-cell imaging, such as time-lapse imaging, FRAP and live correlative light and electron microscopy (CLEM), a method that combines CLEM with live-cell fluorescence imaging (Haraguchi et al., 2008; Asakawa et al., 2012). Using these techniques, we studied conjugating pairs of *T. thermophila* cells that express GFP-tagged nucleoporins and demonstrate that biased *de novo* assembly of MAC-type NPCs in the nuclear envelope (NE) of the anterior nuclei is one of the earliest structural alterations of the post-zygotic nuclei that become committed to MAC differentiation, occurring much earlier than the advent of previously known nuclear differentiation markers, such as nuclear expansion (Rahaman et al., 2008) and nuclear localization of Twi1p (Mochizuki et al., 2002). Our finding suggests that the NPC functions as a master switch to determine nuclear differentiation.

## RESULTS

### Live-cell imaging of the NPC in conjugating *Tetrahymena* cells

To elucidate molecular dynamics and cellular events during the biological processes of conjugation in *T. thermophila* (see Fig. 1A and supplementary material Movie 1), we developed live-cell imaging techniques to observe assembly of nucleoporins (Nups) in pairs of conjugating cells. For this procedure, cells expressing GFP-tagged Nups are induced to conjugate with cells expressing untagged Nups. Consequently, in conjugating pairs, GFP-tagged Nups are pre-assembled in the existing NPCs in the GFP-tagged Nup-expressing cell, whereas in the other, non-expressing wild type cell GFP-tagged Nups are only incorporated into newly assembled NPCs after pair formation. Supplementary material Fig. S1 shows an example for GFP–Seh1, a nucleoporin common to both MAC and MIC NPCs (Iwamoto et al., 2009). Conjugating cells were immobilized in low-melting point agarose (supplementary material Fig. S1A) and observed in the living state using fluorescence microscopy. Because continuous observation was limited to ∼200 min owing to the toxicity of the excitation light (488 nm) to the cells, we acquired observations on separate conjugating pairs and successfully followed the process of sexual reproduction from the crescent MIC stage (meiotic prophase) (0 min in supplementary material Fig. S1B) to the development of the new MAC/MIC (stage 12 in Fig. 1A).

GFP–Seh1 stained both the MAC and MIC in the GFP–Seh1-expressing cell of the conjugating pair (upper partner in supplementary material Fig. S1B), whereas in the non-expressing cell GFP–Seh1 stained only the MIC and not the MAC (lower partner in supplementary material Fig. S1B). This result indicates that during sexual reproduction, GFP–Seh1 expressed in conjugating cells is incorporated into newly assembling NPCs of nuclei derived from the parental MIC, but not into the pre-existing NPCs of the MAC.

### The common Nups are unevenly distributed in daughter nuclei prior to nuclear differentiation

Time-lapse imaging of a conjugating pair of cells from pronuclear exchange to the second post-zygotic division (PZD) is shown in Fig. 1. GFP–Seh1 was segregated approximately equally to the daughter nuclei in the first PZD (12–27 min in Fig. 1B). However, during anaphase of the second PZD, GFP–Seh1 became unequally distributed on the dividing dumbbell-shaped nuclei, showing slightly enriched localization in the anterior side (54 min in Fig. 1B). Then, at the end of the second PZD, several clustered regions of GFP–Seh1 fluorescence were observed in both the anterior and posterior nuclei (0 min in Fig. 1C,D). Over time, GFP–Seh1 distribution became more uniform in the anterior nuclei, but not in the posterior nuclei (0–21 min in Fig. 1C and 0–24 min in Fig. 1D). This localization profile is not unique to GFP–Seh1, but was also observed with Nup93, another common nucleoporin (Fig. 1E). The fluorescence clusters in the anterior nuclei, which are destined to become the new MACs, disappeared by ∼12 min after the end of the second PZD (Fig. 1D). This timing corresponded to the time when the new MACs started to increase in size. In contrast, in the posterior nuclei, which are destined to become the new MICs, GFP–Seh1 remained clustered even after the new MAC had expanded in size (60 min in Fig. 1C). The dynamic changes of localization of NPCs from the end of the second PZD (stage 10 in Fig. 1A) to the development of the new MAC/MIC (stage 12 in Fig. 1A) are summarized in Fig. 1F. Taken together, our results indicate that the distribution of newly assembled NPCs becomes biased in favor of the anterior daughter nuclei during anaphase of the second PZD.

### Assembly of MAC-specific Nup98s prior to nuclear differentiation

The zygotic nucleus is derived from the parental MIC nuclei and, thus, is likely to contain MIC-type NPCs. Consequently, post-zygotic nuclei destined to become MACs should start out with MIC-type NPCs, including MIC-specific Nup98s; therefore, MIC-specific NPCs need to be replaced by MAC-specific NPCs during MAC differentiation. To understand the timing of subtype switching of nucleus-specific Nup98s, we examined the dynamic behavior of mCherry–Nup98 homologs. Twi1p–GFP was also examined as an early marker of nuclear differentiation because it must be transported into the MAC-destined nuclei prior to or during nuclear differentiation: Twi1p is an argonaute family protein required for rearrangement of the MAC genome (Mochizuki et al., 2002).

**Fig. 2.**
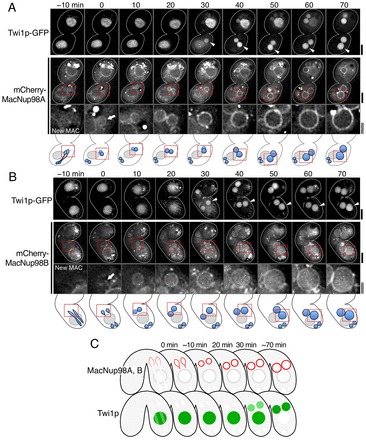
**Dynamic behavior of MAC-specific Nup98s during nuclear differentiation.** (A) Time-lapse images of a conjugating pair of cells expressing mCherry–MacNup98A and Twi1p–GFP from the second PZD (stage 9 in Fig. 1A) to MA-I (stage 11 in Fig. 1A) (*n* = 6 pairs). 3D images (28 *z*-stacks×0.5 µm intervals) were taken every 10 min. The images were processed by reducing background noise and deconvolution. Single focal plane images at the selected time points are shown. Time 0 represents the end of the second PZD. Arrowheads indicate one of the new MACs in which Twi1p–GFP signals appear. Enlarged views of this nucleus are shown in the lower panels. An arrow indicates the anterior nucleus immediately after fluorescence signals of mCherry–MacNup98A start to appear. Scale bars: 10 µm (thick line) and 3 µm (double lines). The magnified regions are the boxed areas in the middle panels and the schematic illustration shown immediately below A. (B) Same as A, except that cells expressing mCherry–MacNup98B, instead of mCherry–MacNup98A, were used and 3D images (32 *z*-stacks×0.5 µm intervals) were taken (*n* = 6 pairs). (C) Schematic diagram of the distribution of MAC-specific Nup98 nucleoporins (red) and Twi1p (green). Time represents the time after the end of the second PZD.

Time-lapse imaging revealed that Twi1p–GFP initially localized to the old MAC until 20 min after the end of the second PZD (−10–20 min in Fig. 2A,B), and then transported into the anterior post-zygotic nuclei at 30 min after the second PZD (see arrowheads in Fig. 2A,B) as the anterior nuclei became larger in size. Nuclear translocation of Twi1p–GFP seems to be attained via the cytoplasm (see 30 min in Fig. 2A,B) as described previously (Mochizuki, 2012). In contrast to Twi1p–GFP, mCherry–MacNup98A and mCherry–MacNup98B appeared in the NE of the anterior nuclei during or immediately after the second PZD (Fig. 2), 30 min prior to the translocation of Twi1p into the MAC-destined nuclei. MacNup98A localized mostly to the NE (Fig. 2A), whereas MacNup98B localized to both the NE and the nucleoplasm (Fig. 2B). We also used GFP-tagged histone H1, a protein that is transported only into MAC (White et al., 1989; Iwamoto et al., 2009), as a marker for nuclear import, and found that histone H1–GFP was transported only into the anterior nuclei (data not shown), suggesting that the anterior nuclei gain the functional MAC-type NPCs. These results indicate that the MAC-type NPCs were assembled on the NE of the anterior nuclei prior to the onset of nuclear transport of Twi1p (summarized in Fig. 2C).

During time-lapse imaging, we observed rare events of rapid disassembly of mCherry–MacNup98A from the NE to the nucleoplasm in the developing MAC in a couple of pairs. These rare events are likely to be abnormal inasmuch as, in these nuclei (arrowheads in supplementary material Fig. S2), Twi1p–GFP initially appeared and some nuclear enlargement occurred, but then Twi1p disappeared and the nuclei failed to fully enlarge (supplementary material Fig. S2). These results argue that assembly of MAC-specific NPCs is required for correct nuclear transport of Twi1p and is a crucial factor for nuclear differentiation. Indeed, acquisition of MAC-type NPCs is the earliest known indicator of MAC differentiation.

### Dynamics of MIC-specific Nup98s during nuclear differentiation

**Fig. 3.**
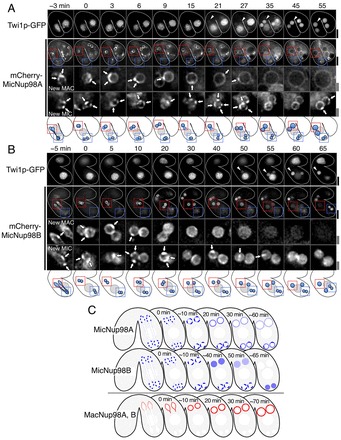
**Dynamic behavior of MIC-specific Nup98s during nuclear differentiation.** (A) Typical time-lapse images of a conjugating pair of cells expressing mCherry–MicNup98A and Twi1p–GFP at stages from the second PZD (stage 9 in Fig. 1A) to MA-I (stage 11 in Fig. 1A) (*n* = 7 pairs). 3D images (40 z-stacks×0.5 µm intervals) were taken every 3 min. The images were processed by reducing background noise and deconvolution. Single focal plane images at each time point are shown. Time 0 represents the end of the second PZD. Arrowheads indicate one of the new MACs in which Twi1p–GFP signals appear; enlarged views of this nucleus and a posterior nucleus are shown in the ‘mCherry–MicNup98A’ middle panels (new MAC) and bottom panels (new MIC), respectively. Note that the top panels of ‘mCherry–MicNup98A’ only show the focus planes with new MACs and, thus, new MICs are not present in these images. The arrows indicate cluster of fluorescence signals of mCherry–MicNup98A in an anterior nucleus and a posterior nucleus, respectively. Scale bars: 10 µm (thick line) and 3 µm (double lines). The magnified regions of the anterior and posterior nuclei are indicated by red and blue squares, respectively, both in fluorescence images and in the schematic illustration immediately below A. (B) Same as A, except that cells expressing mCherry–MicNup98B, instead of mCherry–MicNup98A, were used (*n* = 6 pairs). (C) Schematic diagrams of the distribution of MIC-specific Nup98 nucleoporins (blue in top two drawings) and MAC-specific Nup98s (red in the bottom drawing). Time represents the time after the end of the second PZD.

MIC-specific Nup98s, MicNup98A and MicNup98B, were located in all MIC-derived nuclei from the crescent MIC stage to the nuclear differentiation stage (see blue-colored nuclei in Fig. 1A) and were lost from MAC-destined nuclei by macronuclear anlagen development stage II (MA-II, defined by Martindale et al., 1982; Fig. 1A). To accurately determine when the MAC-destined post-zygotic nuclei lose their MIC-type NPCs, we observed the dynamic behavior of MicNup98s in living cells from the second PZD stage to the MA-I stage (see Fig. 1A). Time-lapse imaging showed that mCherry–MicNup98A fluorescence was evenly distributed to the anterior and posterior daughter nuclei during the second PZD (−3 and 0 min in Fig. 3A), with the fluorescence forming clusters on the NE during and just after nuclear division (−3–9 min in Fig. 3A). The clusters of mCherry–MicNup98A remained in the anterior nuclei until 27 min after the end of the second PZD and were then lost from the NE. Notably, the loss of the mCherry–MicNup98A clusters and the onset of nuclear translocation of Twi1p–GFP occurred at about the same time (35 min in Fig. 3A). In the posterior nucleus, however, the clusters of mCherry–MicNup98A remained on the NE until 55 min and Twi1p–GFP was not translocated into these nuclei (Fig. 3A).

Like mCherry–MicNup98A, another MicNup98 homolog, mCherry–MicNup98B, was also distributed equally to the NE of the anterior and posterior daughter nuclei during the second PZD, and formed clusters on the NE of both anterior and posterior nuclei until 10 min after the end of the second PZD (arrows, −5–10 min in Fig. 3B). However, unlike MicNup98A, at ∼20 min MicNup98B localized to the nucleoplasm of the anterior nuclei, and then began to disappear from the nuclei. These results suggest that the pre-existing MIC-type NPCs in the anterior nuclei are gradually lost. Similarly to the loss of the mCherry–MicNup98A clusters from the anterior nuclei, loss of mCherry–MicNup98B from the nuclei and the onset of nuclear translocation of Twi1p–GFP (arrowheads in Fig. 3B) occurred at about the same time. In the posterior nuclei, mCherry–MicNup98B also localized to the nucleoplasm; however, loss of mCherry–MicNup98B from the NE occurred much later in the posterior nuclei than in the anterior nuclei, and it was not lost from the posterior nuclei during the nuclear differentiation stage. It should be noted that expression of mCherry–MicNup98B might be deleterious since its expression often slowed or interrupted nuclear differentiation (see upper mating partner in Fig. 3B), whereas expression of the other Nup98 homologs tagged with mCherry did not exhibit adverse effects. These results suggest that MicNup98B has a distinct function compared with the other Nup98 homologs in nuclear differentiation.

The behaviors of MicNup98s and MacNup98s are summarized in Fig. 3C. The localization patterns of the MicNup98s were distinct from those of the MacNup98s, whose localization patterns were much more uniform than the MicNup98s (compare 10 min in Fig. 2 with 9 min in Fig. 3A or 10 min in Fig. 3B; also see Fig. 3C and supplementary material Fig. S3H,I). This difference in the localization patterns of the MicNup98s and MacNup98s, even when these Nups were present in the same NE, are likely to reflect the fact that MIC-type and MAC-type NPCs exist as separate complexes in the NE.

### Active assembly of NPCs occurs in new MACs but not in new MICs during early nuclear differentiation

To determine whether *de novo* assembly of NPCs occurs during the early stages of nuclear differentiation, we performed FRAP analysis using GFP–Nup93 as a probe: Nup93 is an immobile NPC component in mammalian cells (Rabut et al., 2004) and, thus, its fluorescence should not reappear after photobleaching unless *de novo* NPC assembly occurs. Because *T. thermophila* Nup93 is a component of both MAC and MIC NPCs (Iwamoto et al., 2009), and because it is immobile in vegetatively growing cells (data not shown), it is expected that *de novo* assembly of NPCs, if any, can be detected by FRAP of GFP–Nup93.

**Fig. 4.**
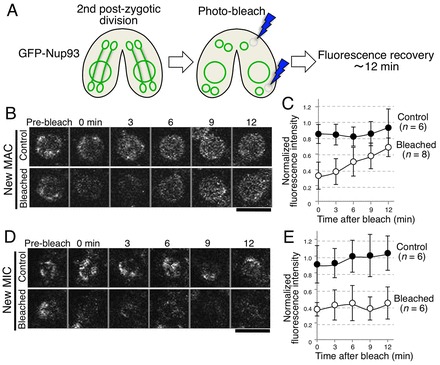
**FRAP analysis during early nuclear differentiation reveals *de novo* NPC assembly in the anterior new MAC but not in the posterior new MIC.** (A) Schematic diagram of this experiment. The stages were monitored by time-lapse observation of GFP–Nup93. At the end of the second PZD, GFP–Nup93 fluorescence in a single nucleus located in the anterior (B,C) or posterior (D,E) region was photobleached, and its recovery monitored for 12 min. (B) A typical example of images for the anterior nucleus (new MAC) is shown. Images of the unbleached nucleus in the same pair are shown as a control. Scale bars: 3 µm. (C) The data of fluorescence recovery were quantified, and the average values obtained from *n* (as indicated) independent experiments are shown. Errors bars indicate standard deviation. (D,E) Same as B and C, except that results for the posterior nucleus (new MIC) are shown, instead of those for the anterior nucleus.

Time-lapse imaging was first carried out to identify the timing of the end of the second PZD before photobleaching. Then, immediately after the end of the second PZD, one of the anterior nuclei or one of the posterior nuclei was photobleached. After photobleaching, fluorescence recovery was monitored for 12 min (Fig. 4A). Fluorescence was recovered in the anterior nucleus (Fig. 4B,C) but not in the posterior nucleus (Fig. 4D,E), suggesting that *de novo* assembly of NPCs occurred in the anterior nuclei (future MAC) but not in the posterior nuclei (future MIC) during this time period. It should be noted that the recovered fluorescence showed a uniform, rather than clustered, distribution on the NE, which correlates well with the uniform distribution of the MAC-type NPCs observed with mCherry–MacNup98s (Fig. 2), rather than the clustered distribution observed with MicNup98s, and suggests that the newly assembled NPCs in the anterior nuclei are MAC-type NPCs.

### Redundant NE and NPC clusters form in early differentiating nuclei

To understand the ultrastructural basis of the distribution of the NPCs on the NE during the early stages of nuclear differentiation, we performed live CLEM on cells undergoing the second PZD. In the live CLEM method, the dynamic behavior of specific molecules of interest in a living cell are first observed by time-lapse imaging using fluorescence microscopy; then cellular structures – such as organelles and membranes – in the same cell are observed using electron microscopy (EM) (Haraguchi et al., 2008; Asakawa et al., 2012).

**Fig. 5.**
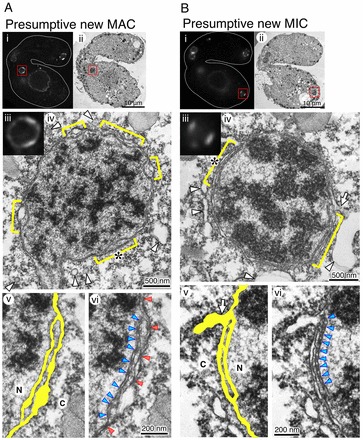
**Redundant NE and NPC clusters found in differentiating nuclei.** Live CLEM was used to observe the ultrastructure of differentiating nuclei; a conjugating pair of cells expressing GFP–Nup93 was monitored using time-lapse imaging, and fixed when the cells reached the end of the second PZD. The fixed specimen was then subjected to electron microscopy. (A) Images of an anterior nucleus (presumptive new MAC). (B) Images of a posterior nucleus (presumptive new MIC). (A,B) (i) A single-section image from the fluorescence *z*-stack of images is shown with a focus on the objective nucleus (boxed red area). (ii) A single-section electron micrograph correlating with the fluorescence image shown in i. (iii) A magnified view of the boxed region in i. (iv) A magnified view of the boxed region in ii. Yellow brackets indicate the regions where NE redundancy is observed. Arrowheads indicate membrane structures near the nucleus. A position where the outer membrane of the NE fuses to another membrane is indicated by an arrow. Asterisks indicate the region where the enlarged view is shown in v and vi. (v, vi) Duplicate images of a magnified view of the region indicated by the asterisk in iv. Yellow areas in v represent the structure of the redundant NE shown in vi. N, nucleoplasm; C, cytoplasm. The arrow in panel v of B indicates a vacuole fusing to the edge of the redundant region. In vi, the positions of the NPCs clustered in the inner (nucleoplasmic side) NE of the redundant NE region are indicated by blue arrowheads, and the NPCs facing the outer (cytoplasm side) NE are indicated by red arrowheads. See also supplementary material Fig. S3A–G.

The conjugating pair of cells that express GFP–Nup93 (shown in Fig. 5) was subjected to live CLEM. The specimen was followed by time-lapse imaging and then fixed with glutaraldehyde at the end of the second PZD for EM imaging. In the anterior nucleus, GFP–Nup93 localized to the NE with weak fluorescence, and in several clusters with strong fluorescence (Fig. 5Ai,iii). CLEM analysis of this nucleus revealed that the NE regions with a strong fluorescence signal had redundant (double) NE with four membranes, whereas the NE regions with a weak fluorescence signal had the usual ‘single’ NE with double membranes (Fig. 5Aiii,iv). In the redundant NE regions, vesicular, cisternal and/or tubular membranes adjoined the NE (indicated by arrowheads in Fig. 5Aiv) and portions of these membranes were often fused to the outer membrane of the NE. These redundant NE structures disappeared after the new MAC grew in size (data not shown). These results suggest that these membranes were probably used as sources of new NE when subsequent nuclear expansion occurred during the generation of the new MAC. A similar redundant NE structure was also observed in the conjugating pair of wild-type cells not expressing any additional Nups (supplementary material Fig. S3A–G).

**Fig. 6.**
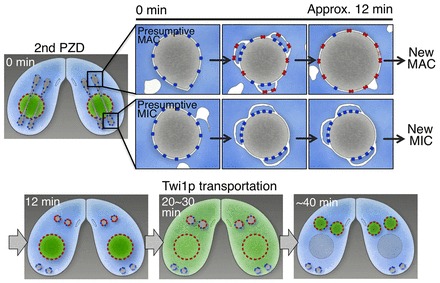
**Schematic summarizing early stages of nuclear differentiation.** Pre-existing MIC-type NPCs (blue dots) are distributed equally to both daughter nuclei during the second PZD, as shown in the 0 min panel. At or immediately after the end of the second PZD, the pre-existing MIC-type NPCs form several clusters on each post-zygotic nucleus. Various membranes, including ER and vacuolar membranes, approach both nuclei and fuse to the NE to generate regions with redundant NE; the MIC-type NPCs are packed in clusters in the inner set of these redundant NEs. Newly synthesized MAC-type NPCs (red dots) assemble on the NE between 0 and 12 min after the end of the second PZD. Importantly, this *de novo* assembly of MAC-type NPCs occurs only in the anterior-localizing presumptive new MACs, but not in the posterior-localizing presumptive new MICs. Size expansion of the presumptive new MACs occurs at ∼12 min after the end of the second PZD. During this event the redundant NEs and NPC clusters in the presumptive new MACs are lost, but the redundant NE and NPC clusters remain unchanged in the presumptive new MICs. Twi1p, a factor required for MAC differentiation, is dispersed into the cytoplasm from the parental MAC and starts to be transported into the new MACs at ∼20–30 min after the second PZD. See also supplementary material Movie 2.

EM images show that the NPCs exist on both of the redundant NEs within the anterior nucleus (Fig. 5Av,vi). The density of the NPCs was significantly higher in the inner NE of the redundant region (nuclear side, 43±27 NPCs per 10 µm of NE length in the EM images) compared with that of the outer NE (cytoplasmic side, 9±10) (*P* = 0.0014, *t*-test) (supplementary material Table S1). The density of the NPCs in single NEs was 17±4. These results indicate that the NPCs are closely packed in the inner NE, and this closely clustered distribution of the NPCs correlates well with the strong fluorescence signals on the NE observed by fluorescence microscopy. This type of strong fluorescence signal (clusters) was observed with common Nups (Seh1 and Nup93) (see −3 min in Fig. 1C and Fig. 1E) and MIC-specific Nups (MicNup98A and MicNup98B) (0–15 min in Fig. 3A, 0–10 min in Fig. 3B, and supplementary material Fig. S3H,I, supplementary material Movie 3), but not with MAC-specific Nups (MacNup98A, and MacNup98B) (0–20 min in Fig. 2A,B, and supplementary material Fig. S3H,I, supplementary material Movie 3), suggesting that the NPCs clustered in the inner NE are pre-existing rather than newly synthesized NPCs (summarized in Fig. 6). In addition, the size of a single NPC in a cluster within the inner NE (51±9 nm in diameter) was significantly smaller than those in other NE areas (63–69 nm) (*P*<10^−6^, *t*-test) (supplementary material Table S1). This result suggests that the clustered NPCs in the inner NE are structurally different from the other NPCs.

In the posterior daughter nuclei (Fig. 5Bi,ii), redundant NEs were also observed (Fig. 5Biii,iv). In these regions, adjoining vesiclular and cisternal membranes (arrowheads in Fig. 5Biv) were fused with the outer NE at portions of these membranes (arrows in Fig. 5Biv,v), and the NPCs were clustered in the inner NE (arrowheads in Fig. 5Bvi). The NE redundancy was more marked in the posterior nuclei than in the anterior nuclei. In contrast to the anterior nuclei, most NPCs were found in the inner NE in clusters (Fig. 5Bvi); only a few NPCs existed in the outer NE of the redundant NEs and in the regions of single NE (Fig. 5Biv,vi and supplementary material Table S1). These NPC-clustered regions corresponded well to the regions where GFP–Nup93 showed a strong fluorescence signal on the NE (compare Fig. 5Biii and 5Biv). The size of a single NPC existing in the inner NE of the posterior nuclei was 53±7 nm, which is significantly smaller than the size of NPCs in other NE areas (∼68 nm) (*P* = 0.0005, *t*-test) and similar to the size of a single NPC in the inner NE of the anterior nuclei (supplementary material Table S1). Since MicNup98A and MicNup98B cluster in the NEs of both anterior and posterior nuclei (Fig. 3), the clustered NPCs in the inner NEs are likely to be MIC-type NPCs (summarized in Fig. 6).

It should be pointed out that, as has already been described by Cole and Sugai (Cole and Sugai, 2012), at the end of the second PZD the chromatin in the anterior nuclei is less condensed than in the posterior nuclei (compare Fig. 5Aiv and 5Biv), suggesting that differentiation can be initiated at this stage. This phenomenon supports the idea that the second PZD is asymmetric and generates differences between anterior MAC-destined, and posterior MIC-destined nuclei.

### Biological significance of a second asymmetric PZD

**Fig. 7.**
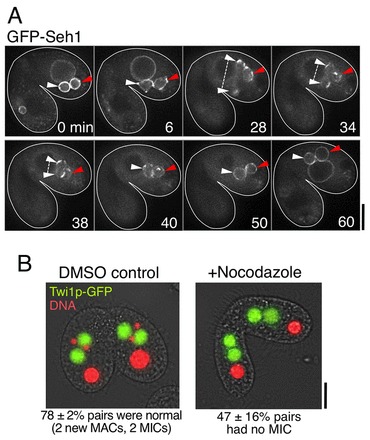
**Post-zygotic nuclei that fail the second PZD differentiate to MACs.** (A) Typical time-lapse images of the second PZD interrupted by nocodazole treatment (*n* = 8 pairs). Cells expressing GFP–Seh1 were induced to undergo conjugation for 6 hrs. Nocodazole was then added to the culture medium at a concentration of 20 µg/ml for 3 hrs. 3D images (28 *z*-stacks×0.5 µm intervals) of GFP–Seh1 were taken manually at intervals of 2–5 min for 60 min; selected time points are shown. Time 0 represents the time when the time-lapse observation started. White arrowheads indicate a nucleus that began to divide (at 28 min), but failed (34–38 min) and reverted back to a single nucleus (at 40 min). Red arrowheads indicate a nucleus that failed to begin dividing (at 6 min). Scale bar: 10 µm. (B) Cells expressing Twi1p–GFP (green) were induced to undergo conjugation for 6 hrs. Conjugating pairs of cells were then treated with 20 µg/ml nocodazole for 3 hrs or DMSO as a control. Cells were fixed with 25% methanol and 4% formaldehyde dissolved in phosphate buffer, and stained with DAPI (red), a DNA-staining reagent. Fifty pairs in stage MA-II were observed under each experimental condition. Scale bar: 10 µm.

Because *de novo* assembly of the MAC-type NPCs on the NE of anterior nuclei starts during anaphase of the second PZD, the second PZD appears to be important for nuclear differentiation. To examine whether the second PZD is, indeed, necessary for MAC differentiation, we performed fluorescence live-cell imaging of GFP–Seh1 in the presence of nocodazole, a tubulin-depolymerizing drug (Fig. 7A). As the second PZD does not occur under this condition, the resulting zygotic nuclei in those cells are products of the first PZD. These nuclei all developed into MACs, generating a cell carrying two new MACs and no MICs in each conjugating partner (Fig. 7A). The new MACs in the amicronucleate cells possessed MAC-type NPCs (supplementary material Fig. S4A) and accumulated Twi1p–GFP (Fig. 7B). These results suggest that the second PZD by itself is not necessary for MAC differentiation, agreeing with previous work (Kaczanowski et al., 1991).

However, previous studies have proposed that the second PZD has a role in positioning the presumptive MICs to the posterior space in close proximity to the cortex, leading to the hypothesis that localization in the posterior region is necessary to escape from a cytoplasmic activity that induces MAC differentiation (Kaczanowski et al., 1991; Gaertig and Cole, 2000). To test whether retention in the posterior region is sufficient for post-zygotic nuclei to differentiate to MICs, we examined whether the nuclear position after the first PZD affects the fate of the post-zygotic nuclei. In the cell shown in supplementary material Fig. S4B, two nuclei moved to the posterior side after the first PZD in the presence of nocodazole and remained there for ∼40 min. Under this condition, both of these posterior-localizing nuclei became MACs and accumulated Twi1p–GFP (supplementary material Fig. S4B). This result suggests that positioning in the posterior region by itself is not sufficient for the post-zygotic nucleus to keep its ‘MICness’, and that MAC differentiation can be triggered even in the posterior cytoplasm. Thus, the cytoplasmic activity that induces MAC differentiation seems to be distributed throughout the cytoplasm and is not limited to the anterior cytoplasm. Taken together, these data suggest that the second PZD is necessary to generate new MICs. However, it is possible that a specific, nocodozole-sensitive microtubule-dependent attachment of the posterior nuclei to the posterior cortex is required to protect the MICs from a uniformly distributed MAC-inducing activity.

## DISCUSSION

The composition of NPCs of MAC and MIC in *T. thermophila* is different (Malone et al., 2008; Iwamoto et al., 2009). MacNup98A and MacNup98B are exclusively found in MAC NPCs, and MicNup98A and MicNup98B are exclusively found in MIC NPCs, whereas other known nucleoporins are common to NPCs of both nuclei (Iwamoto et al., 2009). Thus, MIC-type NPCs containing MicNup98s have to be switched to MAC-type NPCs containing MacNup98s for the presumptive MAC to acquire ‘MACness’ during MAC differentiation. In this study, we demonstrate that *de novo* assembly of MAC-type NPCs has a crucial role within MAC differentiation in *T. thermophila*: Nuclei that do not acquire MAC-type NPCs do not differentiate into MACs. The assembly of MAC-type NPCs is the first known morphological alteration during dimorphic nuclear differentiation in *T. thermophila* and occurs much earlier than the advent of previously known nuclear differentiation markers, such as nuclear expansion (Rahaman et al., 2008) and Twi1p's nuclear localization (Mochizuki et al., 2002).

In addition to *de novo* assembly of NPCs, we also demonstrate that the bias in the assembly of *de novo* NPCs in the anterior and posterior nuclei is crucial in determining the fate of nuclei in *T. thermophila*: *de novo* assembly of NPCs occurs in the anterior nuclei and is prevented from occurring in the posterior nuclei. Masking the posterior nuclei to block *de novo* assembly of NPCs is necessary for dimorphic nuclear differentiation in *T. thermophila*. Biased localization of MAC-type NPCs, however, is not sufficient for irreversible commitment to MAC differentiation. As can be seen in supplementary material Fig. S2, at about 80–90 min after the end of the second PZD, an anterior located, MAC-destined nucleus suddenly loses mCherry–MacNup98A and Twi1p–GFP signals. Therefore, at least one more step in addition to acquisition of MAC-type NPCs might be required for commitment to MAC differentiation. In the ciliate *Paramecium caudatum*, the post-zygotic nuclei undergo three rounds of nuclear division before nuclear differentiation; there, any one of the post-zygotic nuclei selected before the last PZD (i.e. the third PZD in *P. caudatum*) generates a MAC-MIC pair after the last PZD, suggesting that the last PZD is an important step for nuclear differentiation (Mikami, 1980). However, the presumptive MAC nuclei generated at the third PZD in *P. caudatum* can differentiate into a MIC when transplanted into a cell whose MICs were all removed (Mikami, 1985). Thus, in *P. caudatum*, additional events are required after the last PZD for irreversible commitment to MAC differentiation, agreeing with our observation in *T. thermophila* that, after biased acquisition of MAC-type NPCs and initiation of nuclear differentiation, additional events are required for irreversible commitment to MAC differentiation. The findings in *P. caudatum* support our idea of a multiple-step model in nuclear differentiation.

The factors involved in biased assembly of MAC-type NPCs in nuclei that are destined to become MACs remain to be clarified. One option are intra-nuclear factor(s) that can distribute asymmetrically to the daughter nuclei at the second PZD. This hypothesis would explain why zygotic nuclei differentiate into MACs without the occurrence of MIC differentiation when the second PZD is prevented (Fig. 7). Similar asymmetrical nuclear division is observed in the early embryogenesis of *Caenorhabditis elegans*. Biased nuclear localization of β-catenin and other determinants is involved in different cell fates (Sugioka et al., 2011). In *Tetrahymena*, we speculate that the second PZD – which could bring putative intra-nuclear factor(s) to the anterior region – might be important for MIC differentiation rather than for MAC differentiation because, when the second PZD is blocked in response to nocodazole treatment, the nuclei derived from the first PZD usually differentiate into MACs. The mechanisms that localize MAC factors to the anterior region could be important to exclude all of the MAC factors from the posterior region, and this exclusion might be necessary for MIC differentiation.

Intra-cytoplasmic positioning of the nuclei might be another factor that facilitates the biased NPC composition of MAC- and MIC-destined nuclei. Electron micrograph images show that cellular structures were different in the anterior and posterior regions of the conjugating cells at the second PZD: Many more intracellular membrane structures, such as vacuolar vesicles and phagosomes, were present in the posterior region than in the anterior region (Fig. 5Aii,Bii). This non-uniform distribution of intracellular membrane structures may promote the biased NPC composition of MAC- and MIC-destined nuclei. However, we found that posterior-localizing nuclei that arise when a second PZD is prevented following nocodazole treatment can differentiate into MACs (supplementary material Fig. S4B), as reported previously (Kaczanowski et al., 1991). Our results and the study by Kaczanowski et al. indicate that anterior localization is not necessary for MAC differentiation. Nevertheless, the redundant NEs that are present in the posterior nuclei after the second PZD appear to have a role in rendering pre-existing NPCs in these nuclei nonfunctional by packaging them into the inner NE until MAC differentiation has been accomplished (Fig. 5Biv). Thus, the second PZD as well as posterior cytoplasmic positioning both might have a role in preventing the posterior nuclei from acquiring MAC-specific NPCs, thereby, maintaining their MIC character.

NPCs are cellular structures of central importance in nuclear transport. The other key cellular factors involved in the nuclear transport of proteins are the importin family of proteins. It is known that nuclear transport in eukaryotes is mediated by members of the importin β family (Kimura and Imamoto, 2014). Importin β directly binds the cargo protein or interacts with importin α, an adaptor protein that binds to nuclear localization sequences on the cargo and, during transit through the NPC, importin β interacts with constituent nucleoporins. In *Tetrahymena*, 11 members of the importin β family and 13 members of the importin α family have been identified on the basis of sequence similarity (Malone et al., 2008). Whereas most importin β proteins are associated with MAC only, nine importin α proteins are specifically localized to the MIC during vegetative growth and conjugation (Malone et al., 2008). Since nuclear transport of selective cargos, such as Ema1p, Pdd proteins and Lia proteins (Aronica et al., 2008; Madireddi et al., 1996; Nikiforov et al., 1999, 2000; Yao et al., 2007), plays a crucial role in nuclear differentiation, different properties of importin proteins are highly likely to play important roles in establishing and/or maintaining nuclear dimorphism. Clearly, further studies are required to elucidate the functions of importin α and β subtypes during nuclear differentiation in *T. thermophila*.

Our findings here demonstrate that ‘type switching’ of the NPC might lead to differentiation of dimorphic nuclei in ciliates. To resolve the entire process of NPC type switching, further studies are needed. In particular, determining the entire nucleoporin composition of both MAC and MIC NPCs is required as a next step in elucidating type switching of NPCs in *Tetrahymena*.

In summary, sexual reproduction in *Tetrahymena* results in the generation of zygotic nuclei that are derived from the parental MICs. Each of these zygotic nuclei undergoes two nuclear divisions to generate four nuclei in each conjugating cell. Initially, these nuclei all have MIC-type NPCs. Thus, MIC-type NPCs have to be switched to MAC-type NPCs during MAC differentiation. We show that a defining event in MAC versus MIC differentiation is the *de novo* assembly of MAC-type NPCs, occuring exclusively in the presumptive MAC nuclei. Notably, all of the zygotic nuclei have the potential to differentiate into MACs and into MICs. Events probably associated with the second PZD and localization in the posterior region of the cell normally prevent the posterior nuclei from acquiring MAC-type NPCs and differentiating into MACs, thereby maintaining these nuclei as MICs. Our study demonstrates for the first time that type switching of the NPC has a crucial role in the establishment of nuclear differentiation in *Tetrahymena*, and discusses the possibility of NPC differentiation as a master switch in other eukaryotes, when cell fate is determined during differentiation.

## MATERIALS AND METHODS

### *Tetrahymena* cells and culture conditions

CU427 [*chx1-1/chx1-1* (CHX1; cy-s, VI)] and CU428.2 [*mpr1-1/mpr1-1* (mp-s, VII)], were obtained from the *Tetrahymena* Stock Center at Cornell University. Cells were maintained in shallow culture medium containing 1.5% proteose-peptone (Sigma-Aldrich, Tokyo, Japan), 0.5% yeast extract (Sigma-Aldrich) and 0.5% D-glucose, in a plastic dish at 30°C without shaking.

### Induction of conjugation

Cells in logarithmic growth phase were harvested by centrifugation at 700 ***g*** for 1 min, washed twice with starvation medium (40 nM CaCl_2_, 10 mM Tris-HCl pH 7.5), resuspended in starvation medium at 2×10^5^ cells/ml, and then incubated for 12–18 hrs. The starved cells with different mating types were mixed and incubated at 30°C to induce conjugation.

### Expression of fluorescent protein-tagged nucleoporins

Expression of GFP- or mCherry-tagged nucleoporins was induced by addition of CdCl_2_ at 0.5 µg/ml to growing cells or 0.05–0.1 µg/ml to starved and mating cells because the expression is under the control of the Cd^2+^-inducible *MTT1* promoter.

### DNA plasmid construction

cDNAs encoding nucleoporins were amplified by high-fidelity PCR with PrimeSTAR reagent (Takara, Shiga, Japan) from the first-strand cDNAs prepared by using TRIzol reagent (Life Technologies, Tokyo, Japan) according to the manufacturer's protocol. The DNAs were amplified using forward and reverse primers with *Xho*I and *Apa*I sites, respectively. After digestion by *Xho*I and *Apa*I, the PCR products were ligated into the respective restriction sites of pIGF-1, a DNA plasmid used for the exogenous expression of GFP-tagged proteins driven by an *MTT1* promoter in *T. thermophila* (Malone et al., 2005).

To express mCherry-tagged nucleoporins, we first prepared a pICHF-1 vector by replacing the EGFP-coding sequence of pIGF-1 with an mCherry-coding sequence that was codon-optimized for *T. thermophila* (prepared by K. Mochizuki). The pICHF-1 has multi-cloning sites with *Xho*I and *Apa*I at the 3′ end of the mCherry sequence. The cDNAs of nucleoporins were amplified as described above, digested by restriction enzymes, and then ligated into pICHF-1.

Cell strains constitutively expressing Twi1p–GFP are a gift from K. Mochizuki. In these strains, the GFP-coding sequence is fused to the coding sequence of endogenous *TWI1* in the macronucleus using a *neo4* marker cassette. Therefore, expression of Twi1p–GFP is driven by its endogenous *TWI1* promoter.

### Time-lapse imaging of conjugating *Tetrahymena*

Conjugating pairs expressing GFP-tagged protein were suspended in 3% low-melting point agarose (SeaPlaque Agarose; Takara) dissolved in 10 mM Tris-HCl (pH 7.5) at 37°C. Conjugating pair-suspended agarose solution (100 µl) was placed in the center of a glass-bottomed dish and followed by placing a cylinder shaped, flat-soled epoxy resin block (0.95 g weight and 0.44 cm^2^ bottom area) gently on top of the agarose solution. Gelation of agarose was effected by floating the glass-bottomed dish on ice-cold water (within 5 sec) followed by gentle removal of the resin block. This step produced a thin, flat gel in which conjugating pairs were pushed down near to the bottom of the glass. Procedures from suspending the cells in agarose solution to agarose gelation were executed as quickly as possible. The medium used for cell conjugation (100 µl) was layered on top of the agarose gel to prevent the gel from drying out during observation. For microscopic observation, the DeltaVision system (GE Healthcare, Buckinghamshire, England), placed in a temperature-controlled room, was used (Haraguchi et al., 2008). Time-lapse images (16–41 stacks) along the *z*-axis at 0.5–1.5 µm intervals were taken using an oil-immersion objective lens UApo40 (NA = 1.35, Olympus, Tokyo Japan). The images were processed by iterative deconvolution and/or denoising (Boulanger et al., 2009) when necessary.

### Live CLEM imaging

Conjugating pairs of cells expressing GFP–Nup93 were embedded in an agarose gel on a glass-bottomed dish with an addressing grid (grid size, 175 µm) on the coverslip (AGC Techno Glass). Live cells were monitored on the DeltaVision system; to fix cells, 2.5% glutaraldehyde solution was layered over the agarose gel and left for 2 hrs. 3D images (50–60 *z*-stacks×0.2 µm intervals) were obtained using an oil immersion objective lens (PlanApoN60xOSC/NA1.4; Olympus) and processed by deconvolution. EM observation of the same cells was carried out as described previously (Haraguchi et al., 2008): Briefly, the cells in the agarose gel were post-fixed with 1% OsO_4_ (Nisshin EM, Tokyo, Japan) for 1 hr. After sequential dehydration with ethanol, the samples were stained en bloc with 2% uranyl acetate (Wako, Osaka, Japan) for 30 min. The epoxy block containing the cells of interest was trimmed according to the coordinates of the grid and sliced into 80-nm sections. Image data were collected by JEM-2000EX II (JEOL, Tokyo, Japan) with an acceleration voltage of 80 kV.

### FRAP analysis

FRAP experiments were carried out using a confocal microscope (LSM510 META, Carl Zeiss) equipped with a water-immersion objective lens (C-Apochromat 40×, NA = 1.2, Carl Zeiss, Jena, Germany). At 6 hrs after initiation of conjugation GFP–Nup93-expressing conjugating pairs were embedded in an agarose gel on a glass-bottomed dish (as described above). Photobleaching of a defined region (a particular nucleus) of each cell was performed with seven iterations of full power 488-nm light from an argon laser. Image data were collected through a pinhole of 1.42 Airy units (corresponding to an optical section of 1.2-µm thickness) with the 488-nm argon laser at 1.2% of full power.

## Supplementary Material

Supplementary Material
