# Supplementary material for: Biased assembly of the nuclear pore complex is required for somatic and germline nuclear differentiation in Tetrahymena
Source: J Cell Sci. 2015 May 1;128(9):1812–23. doi: 10.1242/jcs.167353 (PMC4432229; doi:10.1242/jcs.167353)
Supplement: Supplementary Material [file supp_128_9_1812__index.html]

Biased assembly of the nuclear pore complex is required for somatic and germline nuclear differentiation in Tetrahymena — Supplementary Material 

# Biased assembly of the nuclear pore complex is required for somatic and germline nuclear differentiation in *Tetrahymena*

## JCS167353 Supplementary Material

**Files in this Data Supplement:**

- **Supplementary Material**
