# Supplementary material for: Biased assembly of the nuclear pore complex is required for somatic and germline nuclear differentiation in Tetrahymena
Source: J Cell Sci. 2015 May 1;128(9):1812–23. doi: 10.1242/jcs.167353 (PMC4432229; doi:10.1242/jcs.167353)
Supplement: Supplementary Material [file supp_128.9.1812_JCS167353.pdf]

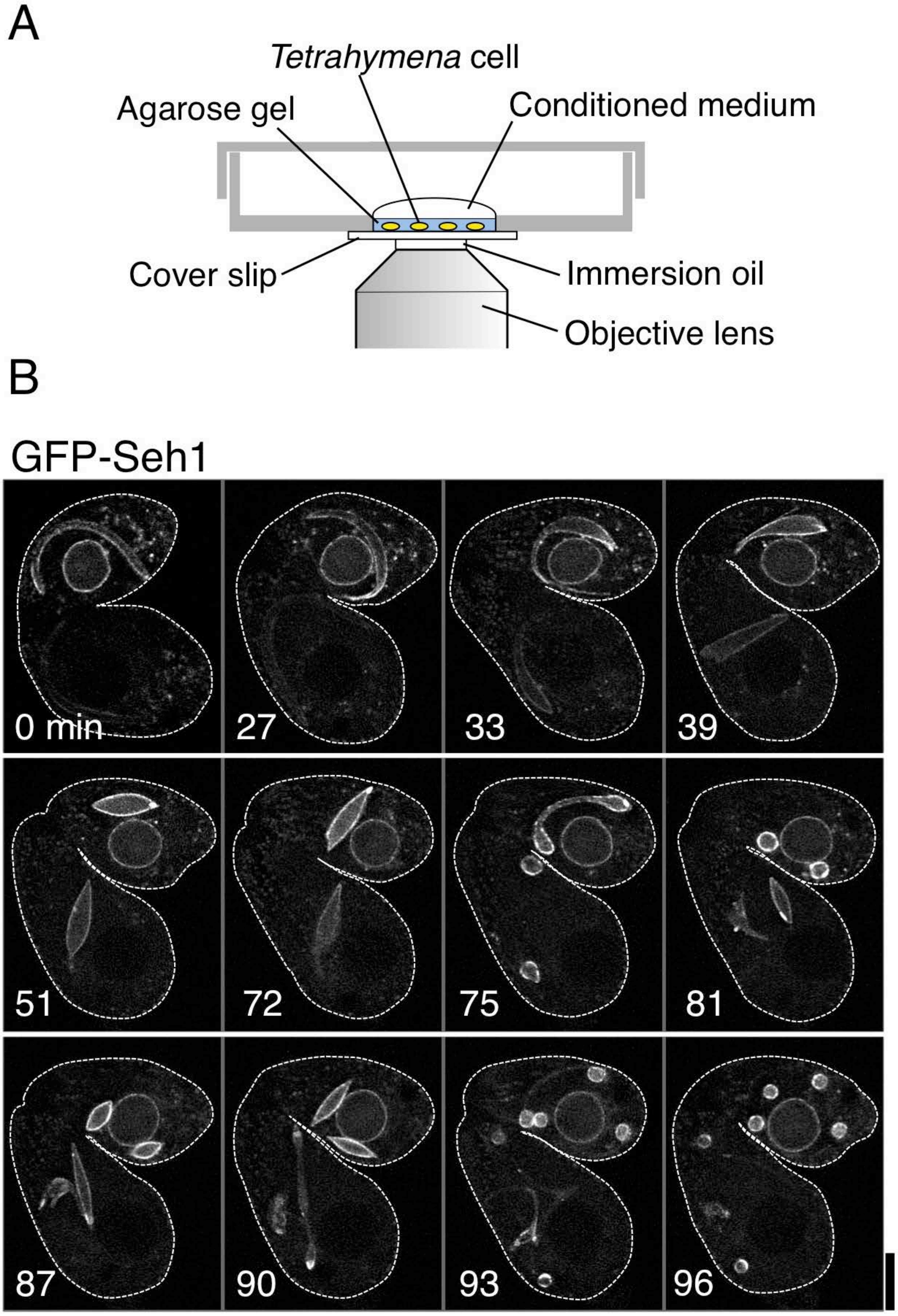

Fig. S1. Time-lapse imaging of nuclear events during conjugation in *Tetrahymena*. (A) Schematic diagram of the set up for live cell imaging using fluorescence microscopy. (B) A GFP-Seh1-expressing cell (upper half) conjugating with a wild-type cell (lower half). Typical images of GFP-Seh1 from the crescent MIC stage to the end of second meiotic division are shown of 16 independent similar observations. The numbers represent the time in minutes after the start of observation. Scale bar, 10 μm.

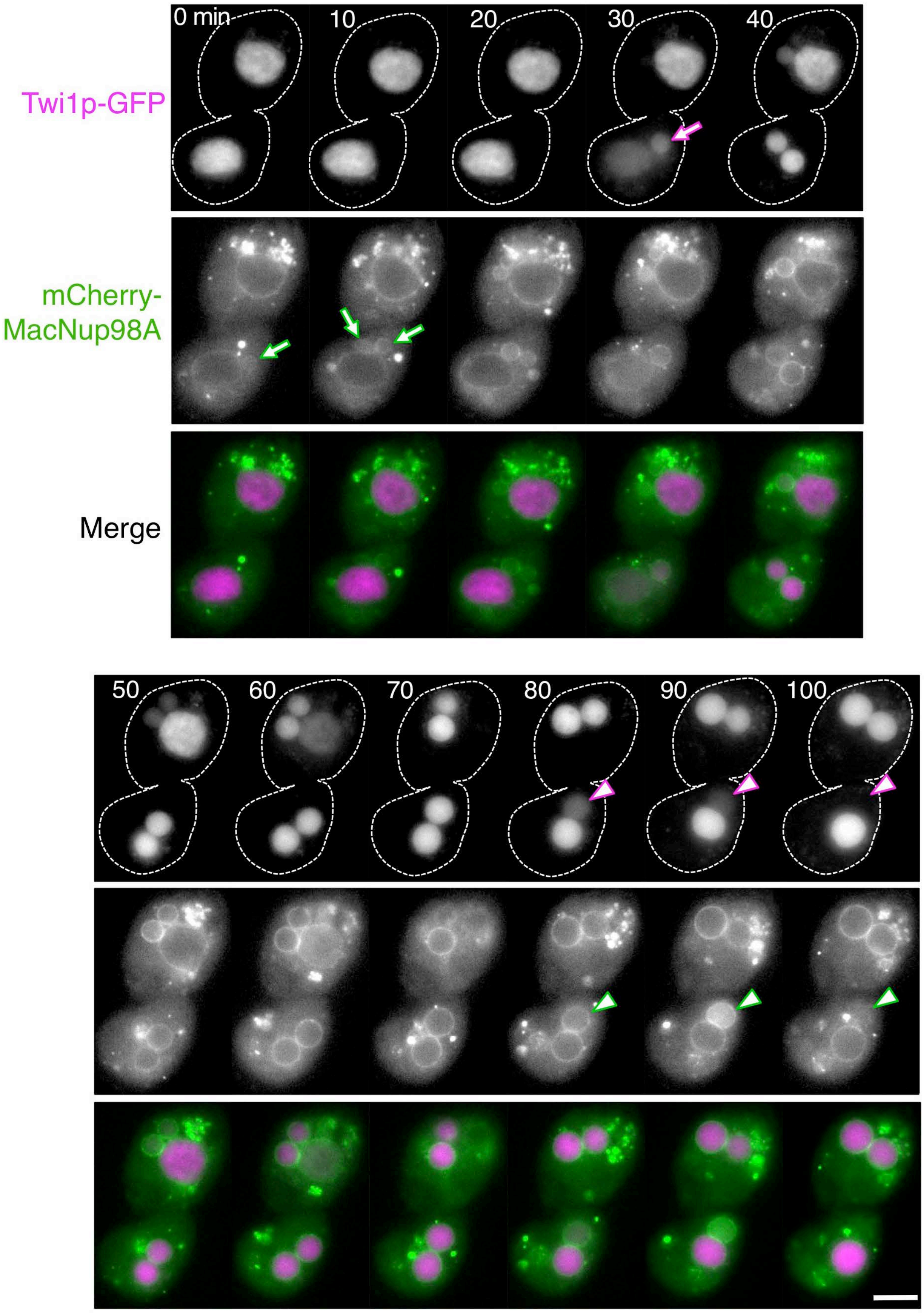

Fig. S2. Abrupt abolishment of MacNup98A assembly and subsequent release of Twi1p from the differentiating MAC. An mCherry-MacNup98A-expressing cell is conjugating with a Twi1p-GFP-expressing cell. Images were taken every 10 min; 28

z-stacks with an interval of 0.5 μm in the z-axis were taken for each time point. The numbers represent the time in minutes after the end of the second PZD. Arrows with green borders indicate the anterior-locating presumptive new MACs in which the NE started to be marked with mCherry-MacNup98A. The arrow with the magenta border indicates a new MAC in which Twi1p-GFP started to accumulate. Arrowheads indicate a developing new MAC that suddenly lost the mCherry-MacNup98A and Twi1p-GFP signals. Scale bar, 10 μm.

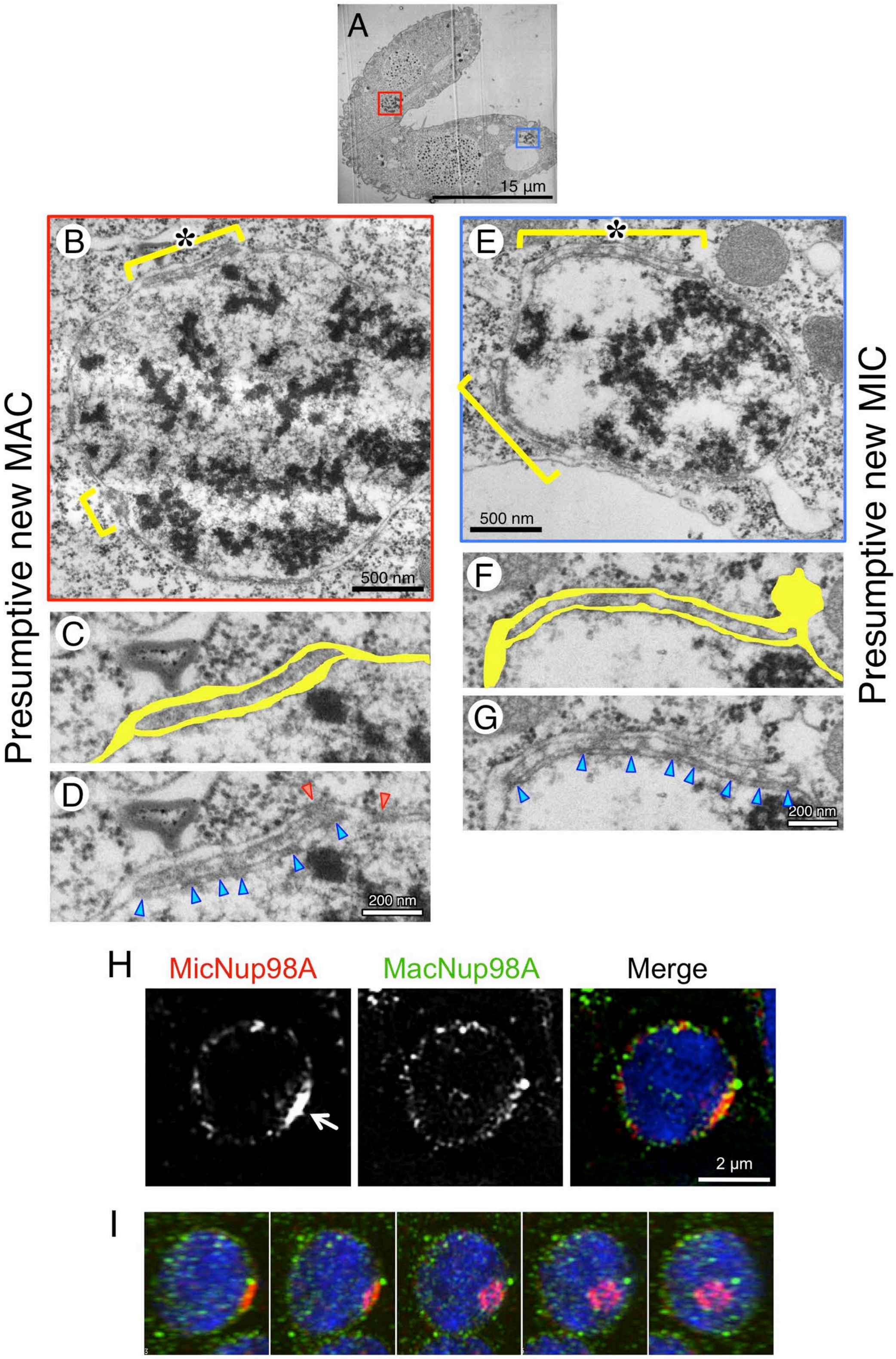

Fig. S3. High-resolution imaging of redundant nuclear envelope and NPC clusters found in differentiating nuclei of a conjugating pair of cells. (A–G) Electron micrographs of a conjugating pair of wild type cells fixed at the end of the second PZD. (A) A single section electron micrograph exhibiting one presumptive new MAC (red-boxed region) and one presumptive new MIC (blue-boxed region). (B) A

magnified view of the presumptive new MAC corresponding to the red-boxed region in A. Yellow brackets indicate the regions where nuclear envelope redundancy is observed. (C, D) Duplicate images of a magnified view of the region indicated by the asterisk in B. Yellow paint in C represents the structure of the redundant NE. In D, the positions of the NPCs in the inner (nucleoplasmic side) NE of the redundant region are indicated by blue arrowheads, and the NPCs in the outer NE (facing the cytoplasm) are indicated by red arrowheads. (E) A magnified view of the presumptive new MIC corresponding to the blue-boxed region in A. (F,G) Duplicate images of a magnified view of the redundant NE region indicated by the asterisk in E. Yellow paint in F represents the structure of the redundant NE. In G, the positions of the NPCs in the inner (nucleoplasmic side) NE of the redundant region are indicated by blue arrowheads. (H, I). Super-resolution fluorescence images of a cluster of the NPC in a presumptive new MAC obtained by three-dimensional structural illumination microscopy (3DSIM). Conjugation was induced between cells expressing mCherry-MicNup98A- and GFP-MacNup98A. Paired cells were fixed with cold methanol, and immunostained with the primary antibodies of anti-RFP mouse monoclonal (MBL) and anti-GFP rabbit polyclonal (Life Technologies), and with the second antibodies of Alexa555-labeled anti-mouse IgG and Alexa488-labeled anti-rabbit IgG (Life Technologies). DNA was counterstained with DAPI. Specimen was mounted in 50% glycerol and observed with a super-resolution microscope system DeltaVision OMX microscope (GE Healthcare) using a custom silicon immersion objective lens 100xUPlanSApo (NA=1.35) (Olympus). 3D images were taken with 0.125-µm intervals along z-direction. (H) A single z-section images of a presumptive new MAC obtained for mCherry-MicNup98A (red), GFP-MacNup98A (green), and DNA (blue), respectively. An arrow indicates a fluorescence-clustered region observed only with MicNup98A. (I) Rotation images of 3D image stacks of the same nucleus as shown in H. Red and green represents mCherry-MicNup98A and GFP-MacNup98A, respectively. See also Movie S3.

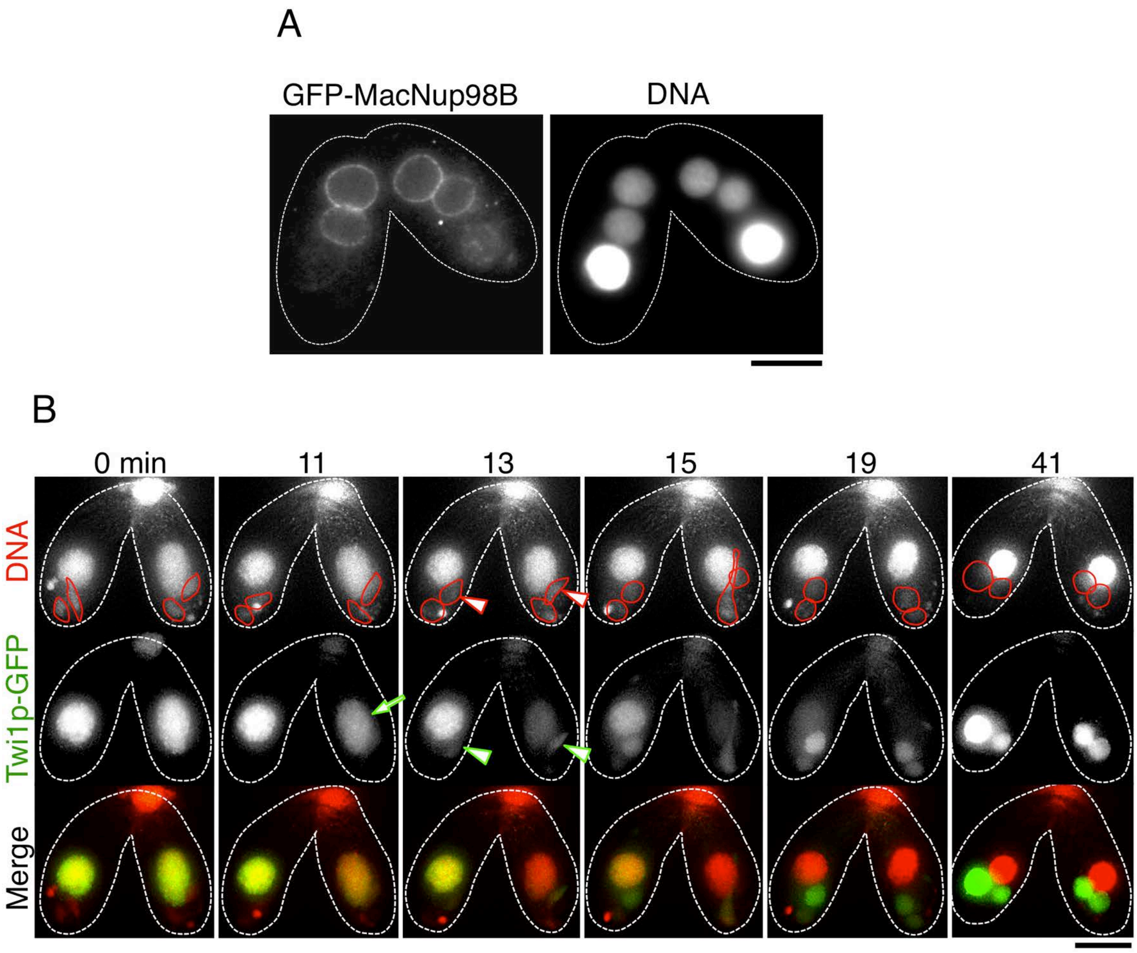

Fig. S4. The asymmetric second PZD is dispensable for MAC differentiation. (A) A conjugating pair of cells expressing GFP-MacNup98B was treated with nocodazole at a concentration of 20 µg/ml for 3 hr and then fixed with a mixture of 25% methanol and 4% formaldehyde in phosphate buffer; the second PZD was disrupted by the nocodazole treatment. The fixed cells were treated with DAPI to stain the DNA and subjected to fluorescence microscopy to obtain GFP and DAPI images. This pair at the MA-II stage has two new MACs and no MIC in each partner; the two developing MACs are marked with GFP-MacNup98B. Scale bar, 10 µm. (B) A conjugating pair of cells expressing Twi1p-GFP was treated with Hoechst33342 to stain the DNA and treated with nocodazole as described in A. Three pairs of cells were then subjected to time-lapse imaging. Similar results were obtained from all of 3 pairs. A typical case is shown. Red and green in the merged images represent DNA and Twi1p-GFP, respectively. Scale bar, 10 µm. Red broken lines in the top panels represent the positions of zygotic nuclei. In this pair, the second PZD was disrupted by nocodazole, generating two post-zygotic nuclei in each half of the pair; these nuclei stayed in the posterior region. The fluorescence signal of Twi1p-GFP accumulated into the nuclei (13–41 min). The strong fluorescence signals in the top region of the DNA panels represent debris unrelated to this pair. Arrowheads indicate the nuclei to which Twi1p-GFP began to be transferred. The green arrow indicates the parental MAC in which the fluorescence Twi1p-GFP signal decreased.

Movie legends

Supplementary Movie 1. Sexual reproduction in *Tetrahymena*

This movie illustrates typical sexual reproduction in *Tetrahymena*.

Supplementary Movie 2. Nuclear differentiation in *Tetrahymena*

This movie illustrates typical nuclear differentiation in *Tetrahymena* from the pronuclear exchange stage (stage 6 in Fig. 1A) to the MA-II stage (stage 12 in Fig. 1A). Red and blue dotty structures represent MAC-type and MIC-type NPCs, respectively. Green represents Twi1p localization. Whitish structures represent tubular, cisternal, or vacuolar membrane structures.

Supplementary Movie 3. A rotation of the 3D image of the NPC clustering structure in the presumptive new MAC

This movie shows a typical structure of the nuclear envelope in presumptive new MAC observed by super-resolution fluorescence microscopy of 3DSIM (see the legend of Fig. S3H,I for details). Red, green and blue colors represent fluorescence of mCherry-MicNup98A, GFP-MacNup98B, and DNA stained with DAPI, respectively. The red regions are corresponding to the region where the NPCs are clustered in the inner nuclear envelope of the redundant nuclear envelope found in the CLEM analysis (Fig. 5), and show a disk-like structure in this movie.

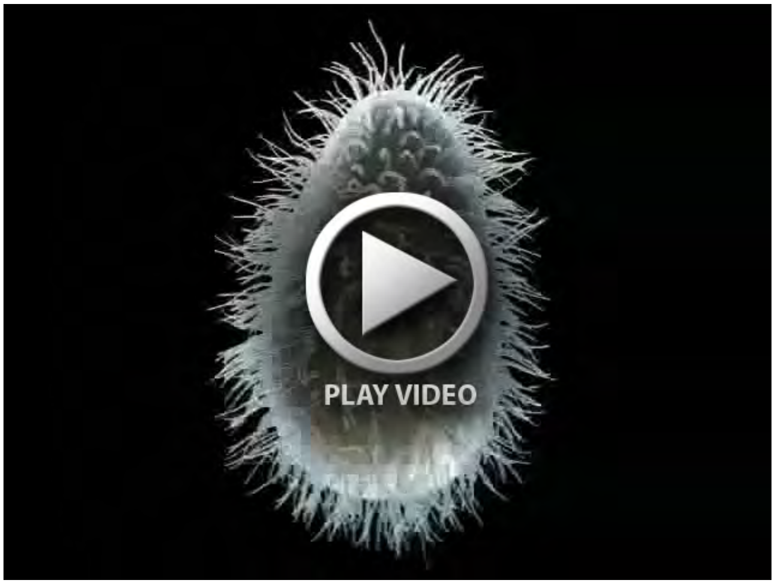

Movie 1.

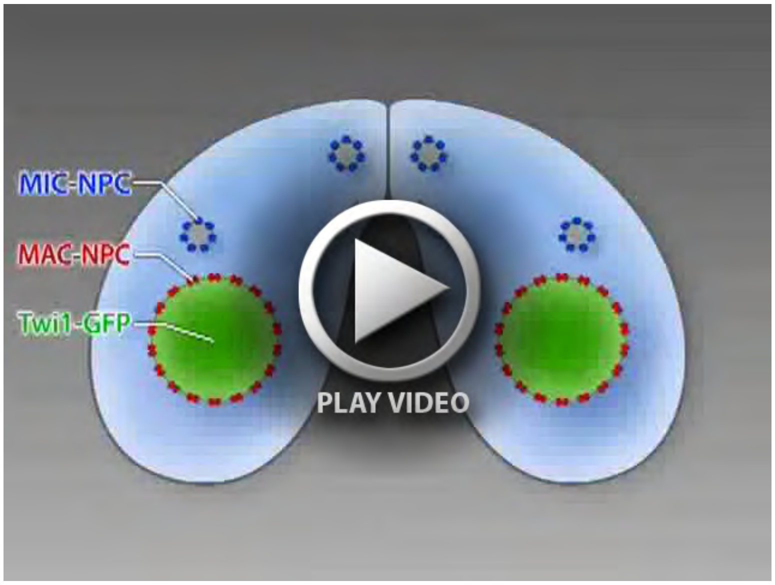

Movie 2.

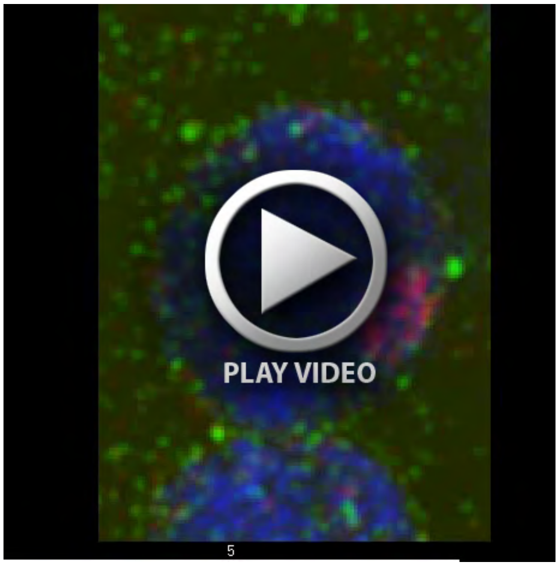

Movie 3.

Table S1. Distribution density and diameter of the NPCs

	NPC number/ 10 m NE	NPC diameter (nm)
**Anterior nuclei (presumptive MAC)**		
Single NE	17 ± 4<br>(total 471,978 nm from 16 nuclei)	68 ± 9<br>(n = 193, from 6 nuclei)
Redundant outer NE	9 ± 10<br>(total 27,746 nm from 13 nuclei)	63 ± 9<br>(n = 25, from 5 nuclei)
Redundant inner NE	43 ± 27<br>(total 24,655 nm from 12 nuclei)	51 ± 9<br>(n = 72, from 5 nuclei)
**Posterior nuclei (presumptive MIC)**		
Single NE	6 ± 6<br>(total 159,403 nm from 11 nuclei)	68 ± 11<br>(n =38, from 6 nuclei)
Redundant outer NE	4 ± 6<br>(total 40,570 nm from 14 nuclei)	68 ± 11<br>(n =12, from 5 nuclei)
Redundant inner NE	57 ± 17<br>(total 40,085 nm from 14 nuclei)	53 ± 7<br>(n = 126, from 6 nuclei)
**Zygotic nuclei**	—	67 ± 12<br>(n = 537, from 10 nuclei)
**Parental MAC**	—	80 ± 11<br>(n = 161, from 6 nuclei)
**MAC of non-paired cell**	—	69 ± 9<br>(n = 199, from 6 nuclei)
**MIC of non-paired cell**	—	64 ± 9<br>(n = 98, from 6 nuclei)

Each value was acquired from TEM images from live CLEM analysis of conjugating pairs: GFP-Nup93-expressing cell × wild-type cell. Wild-type cells were used to obtain the values for starved non-paired cells. The NPC diameter of the parental MAC was measured in the conjugating pairs after fertilization but before Twi1p-GFP transportation.
